# The Habituation Process in Two Groups of Wild Moor Macaques (*Macaca maura*)

**DOI:** 10.1007/s10764-021-00275-7

**Published:** 2022-01-14

**Authors:** Clara Hernández Tienda, Bonaventura Majolo, Teresa Romero, Risma Illa Maulany, Putu Oka Ngakan, Víctor Beltrán Francés, Elisa Gregorio Hernández, Jose Gómez-Melara, Miquel Llorente, Federica Amici

**Affiliations:** 1 Fundació UdG: Innovació i Formació, Universitat de Girona, Pic de Peguera 11, 17003 Girona, Spain; 2 School of Psychology, University of Lincoln, Lincoln, UK; 3 School of Life Sciences, University of Lincoln, Lincoln, UK; 4 Forestry Department, Hasanuddin University, Makassar, Sulawesi Indonesia; 5 Departamento de Antropología Social, Universidad de Sevilla, Sevilla, Spain; 6 Serra Húnter Fellow, Departament de Psicologia, Facultat d’Educació i Psicologia, Universitat de Girona, Girona, Spain; 7 Institut de Recerca i Estudis en Primatologia, Girona, Spain; 8 Research Group Primate Behavioral Ecology, Department of Human Behavior, Ecology and Culture, Max-Planck Institute for Evolutionary Anthropology, Deutscher Platz 6, 04103 Leipzig, Germany; 9 Behavioral Ecology Research Group, Institute of Biology, Faculty of Life Science, University of Leipzig, Leipzig, Germany

**Keywords:** Animal behavior, Wild macaques, South Sulawesi, Group comparison

## Abstract

When studying animal behavior in the wild, some behaviors may require observation from a relatively short distance. In these cases, habituation is commonly used to ensure that animals do not perceive researchers as a direct threat and do not alter their behavior in their presence. However, habituation can have significant effects on the welfare and conservation of the animals. Studying how nonhuman primates react to the process of habituation can help to identify the factors that affect habituation and implement habituation protocols that allow other researchers to speed up the process while maintaining high standards of health and safety for both animals and researchers. In this study, we systematically described the habituation of two groups of wild moor macaques (*Macaca maura*), an Endangered endemic species of Sulawesi Island (Indonesia), to assess the factors that facilitate habituation and reduce impact on animal behavior during this process. During 7 months, we conducted behavioral observations for more than 7,872 encounters and an average of 120 days to monitor how macaque behavior toward researchers changed through time in the two groups under different conditions. We found that both study groups (N = 56, N = 41) became more tolerant to the presence of researchers during the course of the habituation, with occurrence of neutral group responses increasing, and minimum distance to researchers and occurrence of fearful group responses decreasing through time. These changes in behavior were predominant when macaques were in trees, with better visibility conditions, when researchers maintained a longer minimum distance to macaques and, unexpectedly, by the presence of more than one researcher. By identifying these factors, we contribute to designing habituation protocols that decrease the likelihood of fearful responses and might reduce the stress experienced during this process.

## Introduction

Habituation is a learning process by which individuals decrease their response to a stimulus after having been repeatedly exposed to it (Mackintosh, 1987; Stein, 1966; Thorpe, 1963). In animal behavior research, habituation refers to the process by which repeated exposure to humans results in a gradual reduction in animals’ fearful response, until animals no longer perceive humans as a direct threat (Allan *et al.,* 2020; Cipolletta, 2003; Hanson & Riley, 2018; Knight, 2009; McDougall, 2011; Samuni *et al.,* 2014) and/or they treat them as neutral elements of the environment (Magurran & Girling, 1986; Tutin & Fernandez, 1991; Williamson & Feistner, 2011). However, habituation is a multidimensional, mutual, and complex process in which humans and animals continuously and reciprocally adapt to each other (Alcayna-Stevens, 2016; Allan *et al.,* 2020; Ampumuza & Driessen, 2020; Green & Gabriel, 2020; Hanson & Riley, 2018). Through habituation, researchers can spend more time with the study animals and approach them at the distance necessary for detailed behavioral observations. Although telemetry and camera traps may offer appealing alternatives (Boyer-Ontl & Pruetz, 2014; Crofoot *et al.,* 2010; Pebsworth & LaFleur, 2014), habituation often is still necessary to collect detailed behavioral information on the study subjects (Alcayna-Stevens, 2016; Bertolani & Boesch, 2007; Blom *et al.,* 2004; Doran-Sheehy *et al.,* 2007; McLennan & Hill, 2010; Narat *et al.,* 2015; Souza-Alves & Ferrari, 2010; Tutin & Fernandez, 1991; Van Krunkelsven *et al.,* 1999).

Habituation is common in primatology. Through the establishment of several long-term field studies from the 1950s (Knight, 2009; Wilson, 2012; Yamagiwa & Hill, 1998), researchers became aware of the importance of studying animals in their natural habitat to better understand their biology and behavior and to implement effective conservation measures (Crofoot *et al.,* 2010; Rattenborg *et al.,* 2008; Setchell *et al.,* 2016). These studies typically require that the animals first go through a habituation process (Aguiar & Moro-Rios, 2009; Samuni *et al.,* 2014; Williamson & Feistner, 2011), which researchers have described in detail in some cases (Alcayna-Stevens, 2016; Bertolani & Boesch, 2007; Blom *et al.,* 2004; Jack *et al.,* 2008; McLennan & Hill, 2010; Phillips-Conroy, 1998; Raderschall *et al.,* 2011; Tutin & Fernandez, 1991; Williamson & Feistner, 2011). For instance, researchers usually consider primates to be habituated when, over the course of the study, the proportion of time researchers spend with the study subjects significantly increases, but the time required to locate the group and the minimum distance to the researchers decrease (Ando *et al.,* 2008; Blom *et al.,* 2004; Samuni *et al.,* 2014; Williamson & Feistner, 2011). Moreover, researchers consider primates to be habituated, when they progressively increase the likelihood of neutral responses to researchers (i.e., the majority of the group members shows no flee, avoid, threat, or affiliative behaviors toward researchers during the encounter) and decrease fearful responses (e.g., alarm calls; Cipolletta, 2003; Doran-Sheehy *et al.,* 2007; McDougall, 2011; Tutin & Fernandez, 1991; Williamson & Feistner, 2011).

Describing the habituation process is especially important: habituation is a potentially dangerous process, which might posit serious risks to the animals and have significant effects on their welfare and conservation (Fedigan, 2010; Setchell *et al.,* 2016; Williamson & Feistner, 2011). For instance, habituation to researchers might make primates less fearful of humans and thus more vulnerable to disease transmission or poaching in the long term. One recent example is the COVID-19 pandemic, which has raised growing concerns about the risks of transmitting diseases to wild animals (including primates), sometimes with devastating consequences (Damas *et al.,* 2020; Santos *et al.,* 2020). Moreover, habituation might induce changes in nonhuman primate (hereafter, primate) behavior and increase stress levels in the group in the short term (Hockings & Humle, 2009; Knight, 2009; Sak *et al.,* 2013; Shutt *et al.,* 2014; Turner, 2005; Williamson & Feistner, 2011; Woodford *et al.,* 2002). Therefore, when habituation is necessary (e.g., for scientific and conservation purposes), published reports of habituation protocols can at least reveal the factors that a species is particularly sensitive to and inform future habituation efforts (McDougall, 2011; Narat *et al.,* 2015; Samuni *et al.,* 2014). For example, if fearful responses or stress levels in the group increase when researchers approach monkeys in densely forested areas, or when there are more researchers, future studies might consider minimizing the short-term negative effects of habituation by preferentially approaching primates in smaller groups and in open areas. In this way, researchers can implement habituation procedures that likely reduce the stress experienced during the process and thus reduce ethic concerns (Sak *et al.,* 2013; Turner, 2005; Woodford *et al.,* 2002).

During the habituation process, researchers generally agree on the need to avoid sudden movements toward the animals and prefer to gradually approach individuals while avoiding loud noises and gestures (Bertolani & Boesch, 2007; Boesch-Achermann & Boesch, 1994; Williamson & Feistner, 2011). Moreover, a lower number of researchers following the study animals may facilitate habituation (Williamson & Feistner, 2011). However, habituation is a highly complex process which cannot be easily generalized and may differ across species, populations, groups, and even individuals (Allan *et al.,* 2020; Ampumuza & Driessen, 2020), depending on the local and/or specific socio-ecological conditions of the study animals (e.g., previous exposure to humans, degree of frugivory or sociality, home range size, group cohesion, climatic conditions, habitat: Aguiar & Moro-Rios, 2009; Schülke, 2001; Williamson & Feistner, 2011). For example, species that are more arboreal may allow shorter distances to researchers during habituation, because individuals can more quickly flee to a safe place (i.e., in the trees) compared with less arboreal species. In line with this, l’Hoest’s monkeys (*Allochrocebus l’hoesti*) and blue monkeys (*Cercopithecus mitis*) are more cautious when on the ground in the presence of researchers than in trees (Crofoot *et al.,* 2010; Williamson & Feistner, 2011), suggesting that primates may generally perceive trees as a safer place. Moreover, higher visibility may facilitate habituation, by reducing the frequency of sudden encounters and increasing the possibility for animals to scan the area around them (Williamson & Feistner, 2011). For example, habituation of primates in open habitats may be easier than in dense forests (Allan *et al.,* 2020; Ando *et al.,* 2008; Souza-Alves & Ferrari, 2010; Williamson & Feistner, 2011).

Moor macaques (*Macaca maura*) are one of the seven endemic species living on Sulawesi Island (Indonesia) and are classified as Endangered by the IUCN (Lee *et al.,* 2015; Riley, 2010), with their population steadily declining (Lee *et al.,* 2015). Habitat destruction, fragmentation due to agriculture, and urbanization are the main threats to this species, together with hunting and the pet trade (Lee *et al.,* 2015). In the long term, we aimed to establish two new research sites in Sulawesi to collect information on macaque socioecology and behavior and to implement effective conservation measures involving the local communities to protect the macaques (Lee *et al.,* 2015; Setchell *et al.,* 2016). In the short term, we aimed to systematically describe the habituation process of two wild study groups to assess the factors that might influence it and to propose habituation procedures that might minimize stress to the study animals. Although researchers have already investigated some aspects of moor macaque behavior (Hernández Tienda *et al.,* 2021; Lindburg, 1980; Matsumura, 1993, 1996; Matsumura & Okamoto, 1997; Okamoto *et al.,* 2000; Okamoto & Matsumura, 2001; Thierry *et al.,* 2000), there is still a surprising lack of studies describing the habituation process in this genus. One notable exception is a study that analyzed the habituation of a group of moor macaques from an ethnoprimatological perspective, focusing on the intersubjective aspects of the habituation process (Hanson & Riley, 2018). Here, we build on this previous work by (i) providing detailed behavioral information on two other groups of macaques with a different history of exposure to humans, and (ii) conducting a quantitative assessment of some of the factors that can affect habituation (e.g., number of researchers following the study animals, location in trees, visibility).

First, we assessed whether our habituation procedure was effective. In line with previous literature, we predicted that, if habituation took place, the proportion of time with the macaques and the likelihood of macaques’ neutral responses would increase through time, whereas the time to locate the group, the minimum distance, and the likelihood of macaque fearful responses would decrease (Table I). Second, we assessed which factors facilitate the habituation process and reduce behavioral changes in the study groups (i.e., increasing the likelihood of neutral responses and decreasing that of fearful ones; Turner, 2005). In line with previous literature, we hypothesized that conditions that primates are likely to perceive as not threatening would facilitate habituation. Therefore, we predicted that habituation would be facilitated if encounters took place in the presence of fewer researchers (Doran-Sheehy *et al.,* 2007; Iredale *et al.,* 2010), when macaques were in trees (Bertolani & Boesch, 2007; Williamson & Feistner, 2011), in areas with good visibility (Allan *et al.,* 2020; Souza-Alves & Ferrari, 2010), and/or when researchers maintained a larger distance from macaques (Narat *et al.,* 2015; Williamson & Feistner, 2011; Table I). Third, we assessed the consistency of our findings by including two groups of macaques with different previous exposure to humans. Given that previous *nonnegative* exposure to humans may make primates generally less fearful of humans (Sak *et al.,* 2013; Turner, 2005; Woodford *et al.,* 2002), we predicted that the group with more extensive experience with humans could more quickly habituate to the presence of researchers. If previous exposure to humans had been negative, instead, the group might be more fearful of humans and more slowly habituate to the presence of researchers.

**Table I:** Predictions of our study, models used to test them, and whether they were supported

Predictions		Support	Model
1. Proportion of time with macaques increases through time		Yes	1
2. Time to first locate the macaques decreases through time		No	2
Minimum distance to the macaques decreases …	3. ...through time	Yes	3
	6 … with fewer researchers	No	
	7 … with macaques in the trees	No	
	8 … with greater visibility	No	
Neutral group responses increase …	4. ...through time	Yes	4
	6 … with fewer researchers	No	
	7 … with macaques in the trees	Yes	
	8 … with greater visibility	Yes	
	9 … with larger minimum distance	Yes	
Fearful group responses decrease …	5. ...through time	Yes	5
	6 … with fewer researchers	No	
	7 … with macaques in the trees	Yes	
	8 … with greater visibility	Yes	
	9 … with larger minimum distance	Yes	
10. Proportion of time with macaques and neutral group responses increase more quickly in the group with more previous exposure to humans, while time to first locate the macaques, minimum distance to the macaques and fearful group responses decrease more quickly		Partially	1-5

## Methods

### Ethical Note

We took several measures to reduce the likelihood of long- and short-term negative consequences for the macaques. First, researchers never provided food to macaques to facilitate the habituation process. Second, researchers reduced the risk of disease transmission by never approaching macaques closer than 10 m (i.e., minimum safe target distance from the closest member of the group). If macaques approached a researcher within 10 m (7% of total encounters), researchers remained still. If macaques further approached the researchers, trying to come closer than 5 m, the researchers moved away from the animals. Third, throughout the habituation process, researchers stopped approaching the group as soon as an animal showed signs of distress during the encounter (e.g. threatening humans), and retreated to a safer distance. Fourth, our study was completely observational, and we never established any form of physical contact with the macaques. The Indonesian Foreign Research Permit Division, Ministry of Research and Technology, National Research and Innovation Agency, and the Faculty of Forestry of the Hasanuddin University approved the procedures, which also adhered to the American Society of Primatologists Principles for the Ethical Treatment of Nonhuman Primates. Finally, we always prioritized the safety of the study groups and of the researchers conducting the habituation, complying with international laws and regulations (e.g., not allowing foreign researchers in Indonesia). Therefore, we left the field sites at the start of the outbreak of the COVID-19 pandemic for the safety of the researchers and to avoid the risk of disease transmission from humans to the study subjects (Damas *et al.,* 2020; Santos *et al.,* 2020). Since then, local researchers have occasionally monitored the study groups and maintained extensive contact with the local communities. We will resume systematic behavioral observations as soon as Indonesia allows the entrance of foreign researchers.

Despite these precautions to reduce the short-term impact of habituation on the study groups, habituation might still have serious long-term consequences on primates (Sak *et al.,* 2013; Turner, 2005; Woodford *et al.,* 2002). Therefore, researchers should only start habituation after seriously evaluating the advantages and disadvantages it might cause to the study groups. In our case, we considered habituation to be necessary, based on the following considerations. First, we had tried to use alternative methods to initially follow the groups. However, camera traps could not provide detailed behavioral information on the study subjects, and drones also were not effective due to dense vegetation in the study areas. Second, we only initiated habituation after securing long-term funds and human power to ensure a long-term study of the macaques and thus the possibility to continuously monitor activities in the area (e.g., poaching) that would have a negative effect on their conservation. Third, gaining detailed information on the species behavior and socioecology will allow us to better plan and implement effective conservation measures in the future. Finally, we are collaborating with local teachers and institutions to promote knowledge on this endemic species and raise awareness of conservation issues, with potential long-term benefits for the welfare and conservation of this species (Hockings & Humle, 2009; Setchell *et al.,* 2016). None of the authors has a conflict of interest to declare.

#### Data Availability

Data is available on request from the authors.

### Study population and subjects

**Fig. 1:**
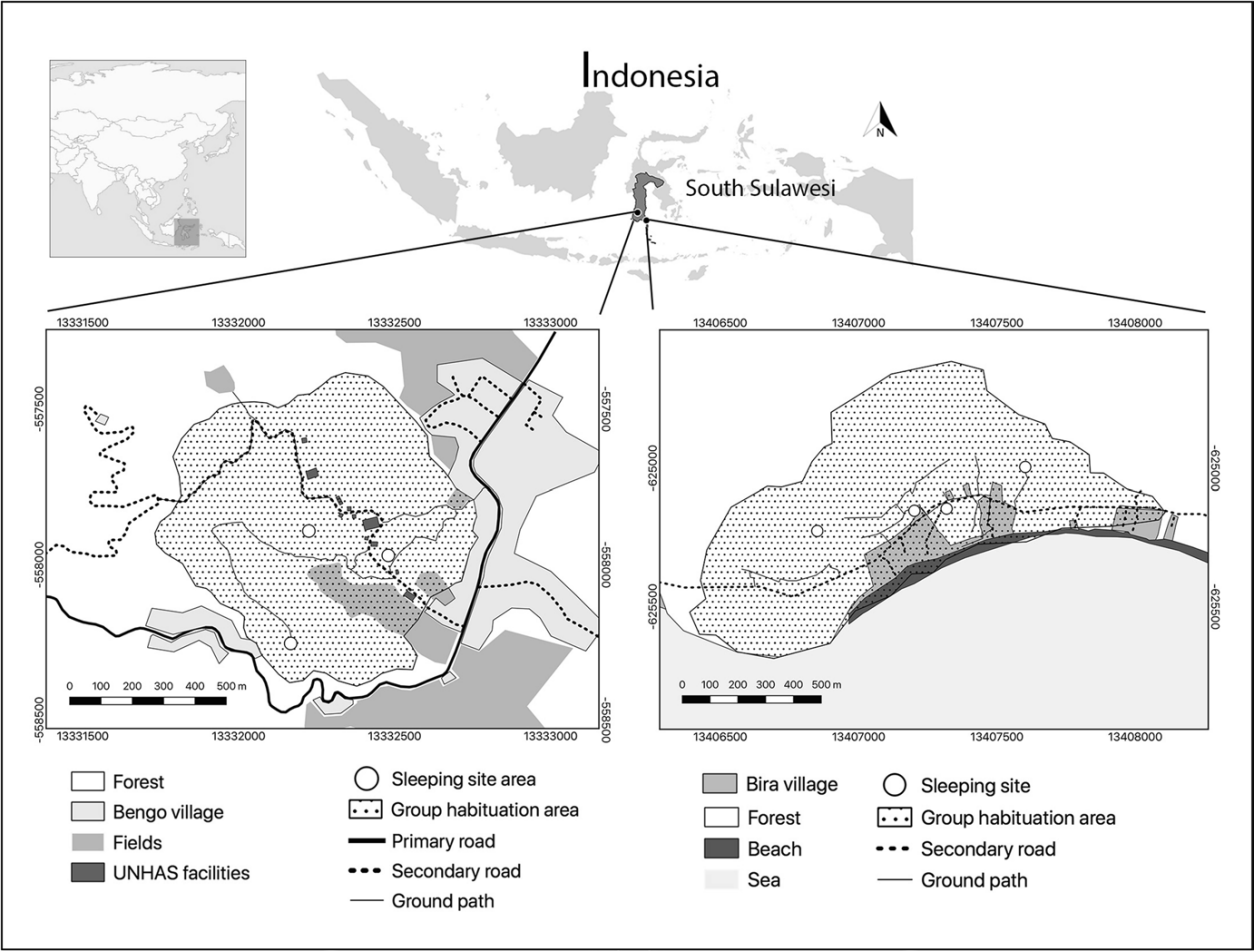
Two field sites in South Sulawesi, Indonesia, where we habituated two groups of moor macaques: Bengo for Merah group (left) and Bira for the Scuba group (right). In each map, we included the main sleeping sites, primary and secondary roads, ground paths, villages and UNHAS (Hasanuddin University) facilities, fields, beach, and forest areas.

We worked on two groups of wild moor macaques from two areas in South Sulawesi, Indonesia (Fig. 1). Both groups live in proximity to other macaque groups, which they occasionally encounter during their daily activities. The first group is located in the Teaching Forest (5°00'S, 119°46'E), in Bengo (Limpoccoe, Cenrana, Maros Regency), a protected area of approximately 1,300 ha managed by the Faculty of Forestry of the Hasanuddin University. The area lies between 400 and 800 m above sea level. It is covered by tropical secondary forest mixed with patches of pines (*Pinus merkusii*) and acacias (*Acacia auriculiformis, A. mangium*) and is marked by two seasons: the wet season (from the end of November until March), and the dry season (from April to November). The study group (hereafter, the Merah group) is located in a forest area, surrounded by the village of Bengo, the facilities of the University of Hasanuddin, rice fields, roads, and paths. Macaques in this area have few interactions with humans, occasionally encountering students collecting ecological data or local villagers living close to the forest. Food resources are abundant in the area; crop foraging is relatively sporadic. At the end of the habituation, the Merah group included 56 individuals: 13 adult males (i.e., older than 6 years), 18 adult females (i.e., older than 4 years), and 25 immatures (including subadults, juveniles, and infants). We collected data on this group from September 2019 until March 2020.

The second group is located in Bara Beach (Pantai Bara, 5°36'S, 120°26'E), close to the village of Bira (Bonto Bahari, Bulukumba Regency). The coast is surrounded by a savannah forest that is considered the last remaining woodland area in South Sulawesi with volcanic stones covered by shrub. The field site is located between 0 and 100 m above sea level. The weather is warm and dry during almost all the year, with a short rainy season (from December to February). The study group (hereafter, the Scuba group) lived in a forest area of approximately 415,000 ha. The urban area is currently expanding, causing fragmentation and habitat loss. The group has access to cultivated fields and to the beach, where most human settlements are found (e.g., hotels and restaurants). Macaques in this area have more frequent interactions with humans, mostly with tourists (mainly national, but also some foreign ones) and local people from the village. The group mostly feeds in the secondary forest and from the fruit trees and crops close to the beach area, although they also collect food from the beach, where tourists might occasionally feed them. The Scuba group included 41 individuals: 6 adult males, 13 adult females, and 22 immatures. We collected data on this group from October 2019 until March 2020.

Researchers determined the composition of both study groups throughout the study by repeatedly counting group members when the groups crossed paths and roads. The number of individuals reported here was the one at the end of the habituation process. During the habituation, however, the composition of both study groups and the number of individuals changed due to migrations, disappearances, deaths, and births, so that the initial number of individuals in the two study groups might have been slightly different. Except for infants, all individuals in both groups were individually recognized at the end of the habituation phase. The number of observation days was similar in both groups (N = 121 for the Merah group, and N = 119 for the Scuba group), and we studied both groups over the same time period. For both groups, researchers collected data 6 days per week, except on days when the weather conditions did not allow researchers go to the field (e.g., heavy rain) or if a researcher felt sick, to avoid disease transmission (Sak *et al.,* 2013). Therefore, we accumulated habituation days over real time in the same way for the two groups. The study ended in March 2020 due to the COVID-19 outbreak when researchers left the field to ensure safety for macaques and researchers. By that time, we considered the habituation process to be over, as both groups could be approached 25 m from the center of the group and individual data could be recorded in a reliable way without fearful or stressful responses by the macaques at a closer distance (i.e., between 25 and 10 m from researchers).

### Procedure and data collection

Researchers followed the macaques during 6-h shifts, either in the morning (from 6 a.m. to 12 p.m.) or in the afternoon (from 12 p.m. to 6 p.m.). The number of shifts varied across days, depending on the availability of the five researchers working at the two field sites and on the climate conditions (66% of the shifts had one researcher, 32% had two, and 2% had three). We chose the time of the shifts so that researchers could start following the macaques when they moved from their sleeping sites and left the forest when macaques returned to their sleeping sites, covering all the activity hours and more easily locating them on the next day. Before the study started, all the researchers were trained on how to habituate macaques, how to estimate distances, and how to record behavioral data. They started data collection only after reaching 90% interobserver reliability.

When researchers were in the field, they always wore the same clothes (a blue t-shirt with the logo of a moor macaque) to maintain the same appearance, facilitate habituation, and increase the chances for macaques to differentiate researchers from other humans (and thus reduce the risks of a broad habituation to humans). At the beginning of each shift, researchers tried to: 1) locate the group, 2) establish visual contact with the group members, 3) record data until visual contact was lost, and 4) follow the group to eventually reestablish visual contact. To locate the group, researchers searched around known sleeping sites and feeding areas with big fruit trees or water supplies (Samuni *et al.,* 2014), listening to their vocalizations and to the songs of the endemic yellow-billed malkoha (*Rhamphococcyx calyorhynchus*)—a bird species that often follows the moor macaques (Strange, 2001). When more than one researcher searched for the group, they followed different paths to locate the group more quickly. Once the group was found, researchers communicated the location of the group by phone and remained together to reduce pressure on the group members.

An encounter started when the group was located and visual contact between researchers and macaques established. As soon as researchers viewed the group, they made a sound (i.e., light whistle or cough) to signal their presence. From that moment, the researchers remained still, making as little noise as possible (Bertolani & Boesch, 2007). The encounter ended when no macaque was visible. If an encounter lasted more than 15 minutes, the researchers tried to move 5-m closer to the macaques (up to the safe minimum distance of 10 m). This strategy was repeated until the distance between researchers and macaques reached 10 m, but immediately stopped if the group appeared stressed. If macaques got closer than 10 m, the researchers remained still. If they further approached researchers (i.e., within 5 m), the researchers stepped back and retreated to avoid zoonotic disease transmission. More than one encounter could happen on the same day.

At the start of each encounter, we recorded the GPS location (Locus Map; Asamm Software, 2009). This allowed us to determine the distribution area of the groups during the habituation process, which might differ from the actual home range of the groups (as during the habituation process researchers might not manage to encounter the macaques in their whole home range, and/or macaques might partly modify their movements due to the presence of the researchers). The maps with the distribution area of the study groups were created in QGIS 3.12.1 (Fig. 1).

Although habituation is a complex and partially subjective process (Allan *et al.,* 2020; Ampumuza & Driessen, 2020; Green & Gabriel, 2020), some observational measures may allow an objective assessment of this process. For each encounter, we therefore noted the following information: 1) habituation day and number of the encounter (for each group, two sequential numbers starting from 1); 2) group ID (i.e., Scuba or Merah); 3) shift (i.e., morning or afternoon); 4) number of researchers (range: 1-3); 5) exact duration of the encounter (in minutes); 6) minimum distance between the researchers and the closest macaque reached during the encounter (in meters); 7) position of the macaques when the encounter started (i.e., whether the majority of visible macaques were on the ground or on trees/high rocks higher than 2 m); and 8) area of the encounter (i.e., where the group was met), categorized as forest area (i.e., tropical secondary forest mixed with patches of pines and acacias for the Merah group and savannah forest with volcanic stones covered by shrubs for the Scuba group), field area (i.e., with crops), beach area (i.e., the shore, excluding resort and hotel facilities), path area (including primary and secondary roads, ground paths, and any open area with a minimum of 1-m width), or human area (i.e., including any other human buildings). When macaques were in trees, researchers prioritized maintaining minimum distance from the animals. Because all researchers were equipped with binoculars, observation conditions also were generally good when macaques were in trees.

During each encounter, and for the whole duration of the encounter, we further assessed macaque behavior in the following way. First, we coded all occurrences of flee (i.e., running away in the opposite direction of the researcher as soon as the encounter started) and avoid behaviors (i.e., interrupting the activity and moving away in the opposite direction; Thierry *et al.,* 2000) happening in the group, as well as threat displays, threat/alarm vocalizations, or affiliative responses (e.g., lip-smacking) toward researchers. Because these behaviors were rather conspicuous and not very frequent, it was possible to reliably detect them whenever they occurred in the group. For each encounter, we then coded as “fearful group response” all of the encounters in which the majority of visible individuals showed flee or avoidance behaviors at least once during the encounter. Moreover, for each encounter, we coded as “neutral group response” all of the encounters in which the majority of visible individuals never showed flee, avoidance, threat behavior, threat/alarm vocalizations or affiliative responses toward the researchers, for the whole duration of the encounter. Finally, once a day, we also recorded: 1) latency to first locate the macaques, as the difference (in minutes) between the time researchers started their shift and the beginning of the first encounter; 2) the total time spent in visual contact with the group, as the sum of the duration of all of the encounters of the day (in minutes); and 3) the total time spent in the forest, as a measure of the daily observational effort (in minutes). While the time to first locate the macaques is a common measure of macaques’ tolerance toward researchers, it also can reflect researchers’ ability to locate the monkeys (Hanson & Riley, 2018).

### Data availability

The datasets generated during and/or analyzed during the current study are available from the last author on reasonable request.

### Data analysis

For all statistical analyses, we used the R software environment R Core Team, version 3.5.0). We used glmmTMB (version 1.0.1; Brooks *et al.,* 2017) to run Generalized Linear Models (GLM) and Generalized Linear Mixed Models (GLMM).

First, we assessed whether our habituation process was effective (i.e., if time spent with the macaques increased through time, while time to locate them decreased) and whether group ID with humans modulated these effects (Models 1 and 2). For this purpose, our unit of analysis was habituation day (N = 240). We entered group ID as a test predictor in both models, so we did not include it as a random factor and used GLMs. In Model 1, we modeled the daily proportion of time spent with the macaques (i.e., daily total time spent in visual contact with the macaque, out of the daily total time spent in the forest, to control for observational effort), using a beta regression distribution to have one data point per day. As test predictors we included the 2-way interaction of group and habituation day to assess whether the daily proportion of time spent with the macaques increased through the habituation process (Prediction 1). Because there often was more than one shift a day (i.e., morning or afternoon), and the number of researchers (i.e., 1-3) could vary between shifts within the same day, these variables were not included in the model. In Model 2, we modeled the time to first locate the group on a day, using a Gaussian distribution. As test predictors, we included the 2-way interaction of group and habituation day to assess whether the time to first locate the group on a day decreased through the habituation process (Prediction 2). As controls, we further included the shift and the number of researchers when the group was first located. In both models, we also included habituation days in which there was no encounter with the macaques. In these cases, we entered 0 as the daily proportion of time spent with the macaques (Model 1) and entered the daily total time spent in the forest as the time to first locate the group (Model 2).

Second, we assessed which factors facilitate the habituation process and reduce behavioral changes in the study groups, and whether the experience with humans modulated these effects (Models 3-5). For this purpose, our unit of analysis was each encounter with the macaques (N = 7,872). We chose “encounter” as the unit of analysis, rather than habituation day, because macaques often responded differently to researchers, even during successive encounters occurring on the same day. As some information was missing in some of these encounters, N varied slightly between models (N = 7,822; N = 7,824; and N = 7,830; respectively). Because we recorded more than one encounter per day, observations were not independent. Therefore, we used GLMMs including habituation day as random factor in all of these models. In Model 3, we modeled the minimum distance to the nearest macaque reached in each encounter, using a Gaussian distribution. As test predictors, we included the 2-way interaction of group and encounter number to assess whether the minimum distance to macaques decreased through the habituation process (Prediction 3). As test predictors, we further included the number of researchers (Prediction 6), the position where macaques were encountered (i.e., ground or tree; Prediction 7) and the area of the encounter (Prediction 8). In Model 4, we modeled as a binomial response the occurrence of neutral group responses (i.e., whether the majority of visible individuals showed no avoidance, flee, threat behavior, threat/alarm vocalizations, or affiliative behaviors to the researchers for all the duration of the encounter). As test predictors, we included the 2-way interaction of group and encounter number to assess whether the occurrence of neutral group responses increased through the habituation process (Prediction 4). As test predictors, we further included the number of researchers (Prediction 6), the position of the macaques (i.e., ground or trees; Prediction 7), the area of the encounter (Prediction 8), and the minimum distance to the nearest monkey reached in each encounter (Predictions 9). Model 5 was identical to Model 4, with the only difference that the binomial response was the occurrence of fearful group responses in each encounter (i.e., whether the majority of visible individuals showed avoidance or flee behaviors at least once during the encounter).

In all models, we further compared the two groups to analyze the effect of group ID on the daily proportion of time spent with the macaques (Model 1), on the time to first locate the group on a day (Model 2), on the minimum distance to the nearest macaque reached in each encounter (Model 3), and on the occurrence of neutral (Model 4) and fearful (Model 5) group responses in each encounter. In particular, we expected the proportion of time spent with macaques and neutral group responses to increase more quickly in the group with more exposure to humans, and time to first locate the macaques, minimum distance to the macaques, and fearful group responses to decrease more quickly (Prediction 10).

For all models, we *z*-transformed continuous predictors to facilitate model convergence and interpretation of model coefficients. We used likelihood ratio tests (Barnett & Dobson, 2018) to compare full models containing all predictors with null models containing only control predictors and random factors. When these comparisons were significant, we further assessed which predictors had a significant effect using the summary function. When 2-way interactions were included in the model, we also always included the main terms. If the 2-way interaction was not significant, we ran the full model again, after removing the interaction and only leaving the main effects. If a categorical predictor with more than two categories was significant, we conducted post-hoc comparisons using Tukey tests with estimate marginal means, using the emmeans package (Estimated Marginal Means, aka Least-Squares Means, version 1.4.5; by Lenth et al., 2022). We reported significant post-hoc comparisons in the Results, including estimates and *P* values. To rule out collinearity, we used the performance package (version 0.4.6; Lüdecke et al., 2020) and determined the variance inflation factors, or VIFs (Field, 2009), which were minimal (maximum VIFs across all models ≤ 2.95). We checked model assumptions and detected no convergence or stability issues in the models presented. Finally, we only included the 2-way interaction of group with time (i.e., habituation day or encounter number) in the models, because we predicted that previous exposure to humans would affect the rapidity with which macaque groups react to humans during the habituation phase (Prediction 10), but not the direction of the effect of the single predictors on their response during the habituation (e.g., macaques with more exposure to humans might more quickly increase their neutral group response through time compared with macaques with less exposure to humans, but in both groups the position of the macaques high in the trees will have a similar positive effect on the occurrence of neutral group responses).

## Results

During the habituation process, the proportion of time spent with the group was $\overline{\mathrm{X}}$ = 191 h for the Merah group (i.e., 21% of the time spent in the forest, range: 0-56%; N = 121 days) and $\overline{\mathrm{X}}$ = 533 h for the Scuba group (i.e., 45%, range: 0-95%; N = 119 days). Encounter duration ranged from 30 s to 15 min. for both groups (Merah: $\overline{\mathrm{X}}$ = 5 min ± SD = 5, N = 2,493; Scuba: $\overline{\mathrm{X}}$ = 6 min ± SD = 6, N = 5,369). The number of encounters in each shift differed in the two groups (Merah: $\overline{\mathrm{X}}$ = 17 ± SD = 12, N = 146; Scuba: $\overline{\mathrm{X}}$ = 27 ± SD = 15, N = 202). Time between encounters was similar in both groups (Merah: $\overline{\mathrm{X}}$ = 11 min ± SD = 28, N = 2,083; Scuba: $\overline{\mathrm{X}}$ = 9 min ± SD = 18, N = 3,691). Time spent to first locate the group on each day ranged from 0 to the whole duration of the shift for both groups (Merah: $\overline{\mathrm{X}}$ = 107 min ± SD = 118, N = 121; Scuba: = 49 min ± SD = 70, N = 119). During the whole habituation process, the minimum distance (±SD) to the closest macaque was 23 ± 10 m for the Merah group, and 17 ± 7 m for the Scuba group. By the end of the study, both groups could be reliably approached to ≤25 m. In particular, the minimum distance reached in all daily encounters was never >25 m in the last 15 observation days for the Merah group, and in the last 24 observation days for the Scuba group.

**Fig. 2:**
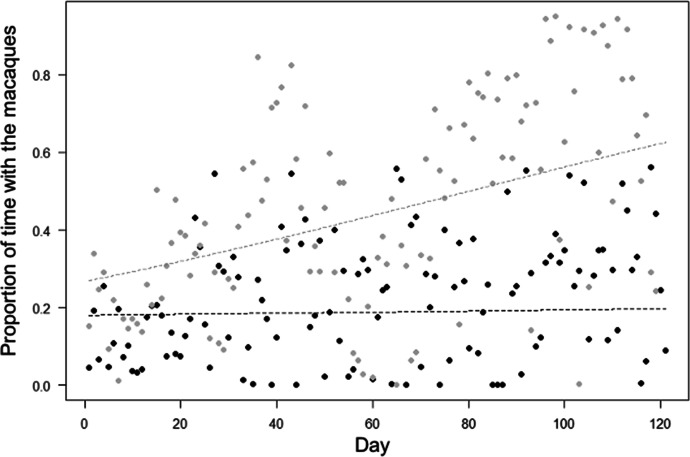
Proportion of time researchers spent with moor macaques in Bengo and Bira on Sulawesi Island, Indonesia (September 2019 to March 2020), as a function of time (i.e., habituation day). Indicated is the proportion of time spent with macaques per observation day (points), and the fitted model (dashed lines). Black represents observations and model line for Merah group, and gray for Scuba group.

Our habituation process was effective (i.e., the proportion of time spent with the macaques increased through time, although time to locate the macaques did not change), and group ID modulated these effects (Models 1-2). For Model 1, the full-null model comparison was significant (LRT: χ^2^ = 77.97, df = 3, *P* < 0.001). The significant 2-way interaction between observation day and group (Table II) suggested that the proportion of time spent with the macaques varied through time in a different way in the two groups. In particular, the proportion of time with the macaques quickly increased through time in the Scuba group, which is more used to encounters with humans from the local communities (in line with Prediction 1; Table I), but remained almost constant in the Merah group, which lived in a forest farther away from human settlements (in line with Prediction 10; Table I; Fig. 2).

**Table II:** Results of models testing the influence of time (i.e. day of habituation, encounter), group ID and other factors (macaque position in the trees, number of researchers, area of the encounter) on the time spent with the macaques, time to locate them, minimum distance between researchers and macaques, and probability of neutral and fearful group responses, in moor macaques in Bengo and Bira on Sulawesi Island, Indonesia (September 2019 to March 2020)

Model	Estimate	SE	z values	2.5% CI	97.5% CI	P
1: Proportion of time with the Macaques (beta-regression)						
Intercept	-1.46	0.11	-13.46	-1.67	-1.25	-
Day	0.03	0.11	0.30	-0.18	0.24	0.767
Group (Scuba)	1.21	0.14	8.52	0.93	1.49	<0.001*
Day * Group (Scuba)	0.41	0.14	2.80	0.12	0.69	0.005*
2: Time to first locate the Macaques (Gaussian)						
Intercept	155.36	19.35	8.03	117.44	193.27	-
Day	4.86	6.43	0.76	-7.73	17.46	0.449
Group (Scuba)	-43.26	13.08	-3.31	-68.89	-17.61	<0.001*
*Shift (morning)*	-92.35	14.80	-6.24	-121.35	-63.35	<0.001*
*Number of researchers*	12.01	13.38	0.90	-14.21	38.22	0.369
3: Minimum distance between researchers and Macaques (Gaussian)						
Intercept	17.33	1.05	16.52	15.28	19.39	-
Encounter	-7.98	0.43	-18.52	-8.82	-7.13	<0.001*
Group (Scuba)	-0.59	0.36	-1.64	-1.29	0.11	0.101
Encounter * Group (Scuba)	4.97	0.38	13.25	4.24	5.71	<0.001*
Encounter area (field)	2.07	1.25	1.65	-0.39	4.52	0.099
Encounter area (forest)	0.03	0.99	0.03	-1.92	1.97	0.979
Encounter area (human)	1.09	1.03	1.05	-0.93	3.10	0.292
Encounter area (path)	1.69	1.02	1.65	-0.32	3.69	0.100
Macaque position (tree)	1.17	0.18	6.49	0.82	1.53	<0.001*
Number of researchers	-0.01	0.23	-0.03	-0.46	0.44	0.975
4: Neutral group responses (binomial)						
Intercept	1.42	0.38	3.78	0.68	2.16	-
Encounter	1.40	0.13	11.15	1.16	1.65	<0.001*
Group (Scuba)	-0.19	0.10	-1.83	-0.39	0.01	0.068
Encounter * Group (Scuba)	-0.98	0.11	-8.71	-1.21	-0.76	<0.001*
Encounter area (field)	-0.74	0.46	-1.62	-1.63	0.16	0.105
Encounter area (forest)	-1.22	0.36	-3.36	-1.93	-0.51	<0.001*
Encounter area (human)	-0.72	0.38	-1.91	-1.45	0.02	0.056
Encounter area (path)	-0.95	0.37	-2.54	-1.68	-0.22	0.011*
Macaque position (tree)	0.46	0.06	8.06	0.35	0.57	<0.001*
Number of researchers	0.38	0.07	5.48	0.25	0.52	<0.001*
Minimum distance	0.29	0.03	9.68	0.23	0.35	<0.001*
5: Fearful group responses (binomial)						
Intercept	-0.76	1.20	0.63	-1.59	3.11	-
Encounter	-1.19	0.12	-9.62	-1.43	-0.95	<0.001*
Group (Scuba)	-0.12	0.10	1.17	-0.08	0.32	0.244
Encounter * Group (Scuba)	0.83	0.11	7.47	0.62	1.05	<0.001*
Encounter area (field)	0.54	0.46	1.19	-0.35	1.44	0.235
Encounter area (forest)	1.03	0.36	2.83	0.31	1.74	0.005*
Encounter area (human)	0.52	0.38	1.39	-0.21	1.26	0.164
Encounter area (path)	0.77	0.37	2.08	0.04	1.50	0.038*
Macaque position (ground)	-2.06	1.14	-1.81	-4.29	0.18	0.071
Macaque position (tree)	2.54	1.14	-2.23	-4.78	-0.31	0.026*
Number of researchers	-0.39	0.07	-5.44	-0.52	-0.25	<0.001*
Minimum distance	-0.29	0.03	-9.57	-0.35	-0.23	<0.001*

Control predictors in italics. In parentheses, we indicate the reference category. *Significant *P* values for the test predictors (in bold).

**Fig. 3:**
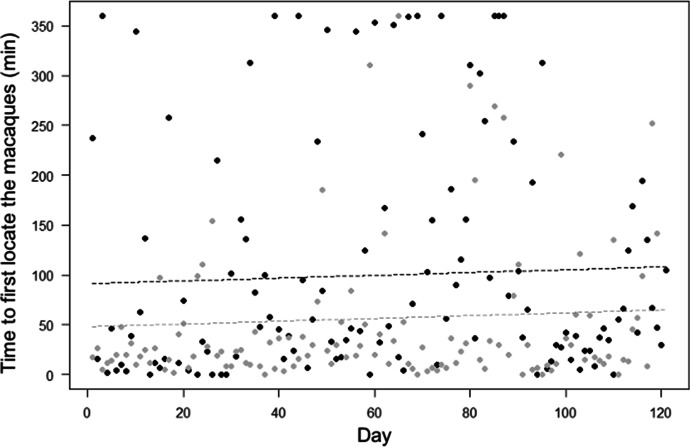
Time required to first locate moor macaques (in minutes) in Bengo and Bira on Sulawesi Island, Indonesia (September 2019 to March 2020), as a function of time (i.e., habituation day). Indicated is the time required to first locate macaques per observation day (points) and the fitted model (dashed lines). Black represents observations and model line for Merah group, and grey for Scuba group.

The full-null model comparison was also significant for Model 2 (LRT: χ^2^ = 10.84, df = 3, *P* = 0.013). The time required to first locate the macaques, however, did not decrease through time (in contrast with Prediction 2; Table I), but was generally higher in the Merah than in the Scuba group (partially in line with Prediction 10; Tables I and II; Fig. 3).

**Fig. 4:**
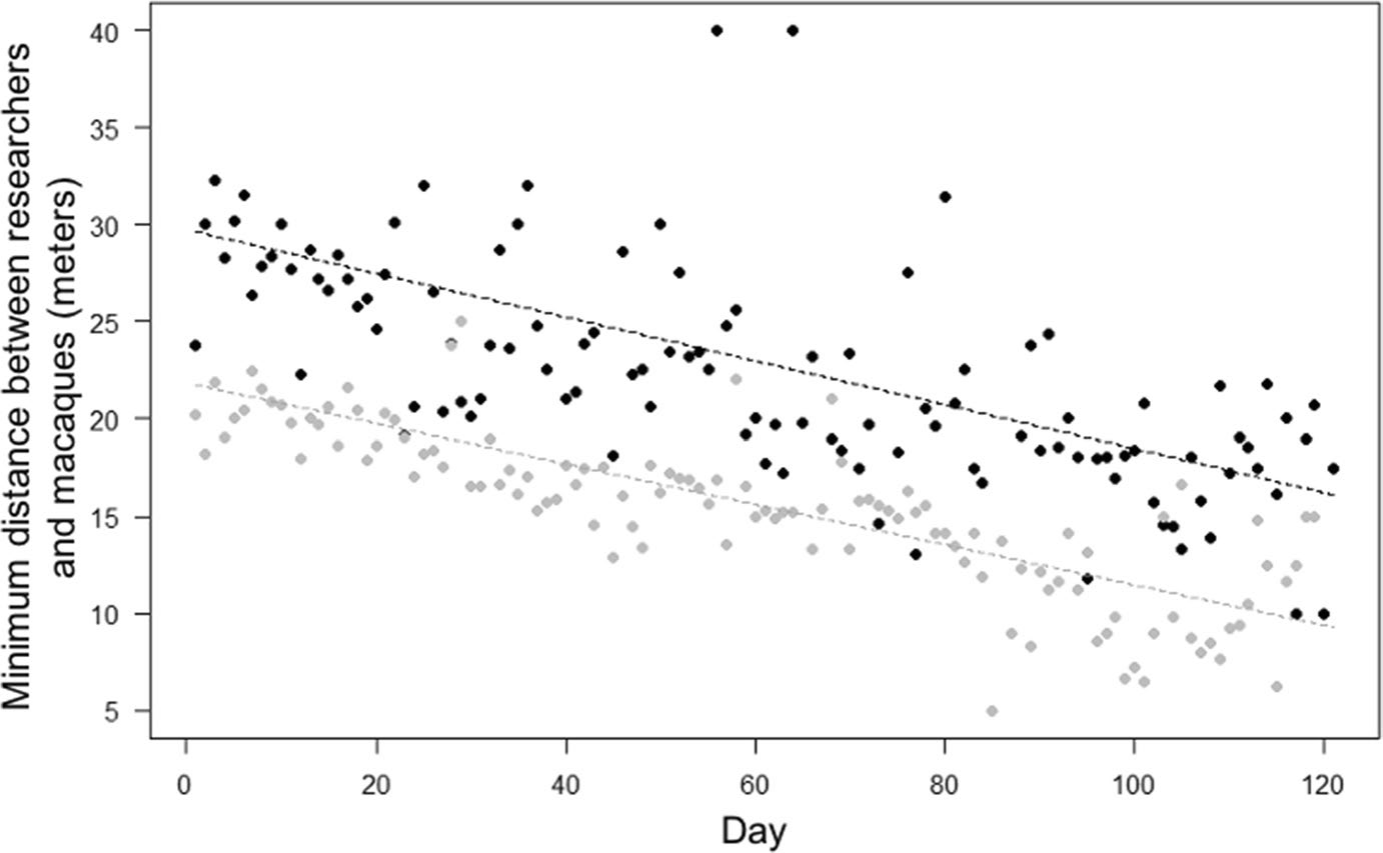
Minimum distance between researchers and moor macaques, in Bengo and Bira on Sulawesi Island, Indonesia (September 2019 to March 2020), as a function of time (i.e., observation day). Indicated is the mean of minimum distance across encounters per observation day (points), and the fitted model (dashed lines). Black represents observations and model line for Merah group, and grey for Scuba group. We aggregated data points per observation day for clarity, so the model depicted here differs from Model 3 (where encounter number instead of day number was used as test predictor). The model is an interaction plot (between day and group).

The habituation process was facilitated by several factors, and group ID partially modulated these effects (Models 3-5). In particular, the full-null model comparison was significant for Model 3 (LRT: χ^2^ = 1443.20, df = 9, *P* < 0.001). The 2-way interaction between encounter number and group was also significant (Table II), showing that the minimum distance between macaques and researchers significantly decreased through time in both groups (in line with Prediction 3; Table I), but more quickly in the Merah than in the Scuba group (in contrast with Prediction 10; Table I; Fig. 4). The number of researchers (Prediction 6) had no significant effect on the minimum distance reached during the encounter (Table II). However, the position of the macaques in trees predicted a larger minimum distance to the researchers (in line with Prediction 7; Tables I and II). The area of the encounter also did not predict the minimum distance reached (Prediction 8).

**Fig. 5:**
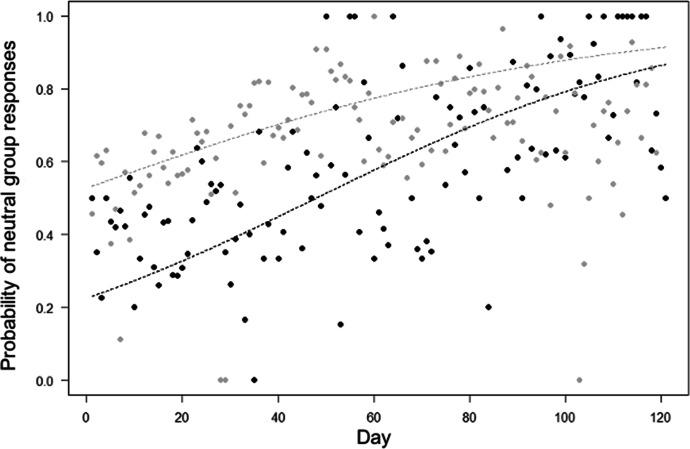
Mean probability that moor macaques in Bengo and Bira on Sulawesi Island, Indonesia (September 2019 to March 2020) displayed neutral group responses during the encounter, as a function of time (i.e., observation day). Indicated is the mean probability of showing neutral responses per observation day (points) and the fitted model (dashed lines). Black represents observations and model line for Merah group, and grey for Scuba group. We aggregated data points per day for clarity, so the model depicted here differs from Model 4 (where encounter number instead of day number was used as test predictor). The model is an interaction plot (between day and group).

For Model 4, the full model significantly differed from the null one (LRT: χ^2^ = 454.33, df = 10, *P* < 0.001). The probability of neutral group responses increased through time in both groups (in line with Prediction 4; Tables I and II), but more quickly in Merah than in Scuba group (in contrast with prediction 10; Table I; Fig. 5). Moreover, neutral group responses were more likely when more researchers were present (in contrast to Prediction 6), and when macaques were in trees (in line with Prediction 7). The probability of neutral group responses varied depending on the area of the encounter (in line with Prediction 8; Tables I and II), being higher in areas frequently used by humans (estimate: 0.79), in paths (estimate: 0.75) and beach areas (estimate: 0.88), compared with forest areas (estimate: 0.69; *P* values for comparisons, respectively: *P* < 0.001; *P* = 0.020; *P* = 0.007). Finally, the probability of neutral group responses was higher when researchers maintained a larger minimum distance to macaques (in line with Prediction 9; Tables I and II).

**Fig. 6:**
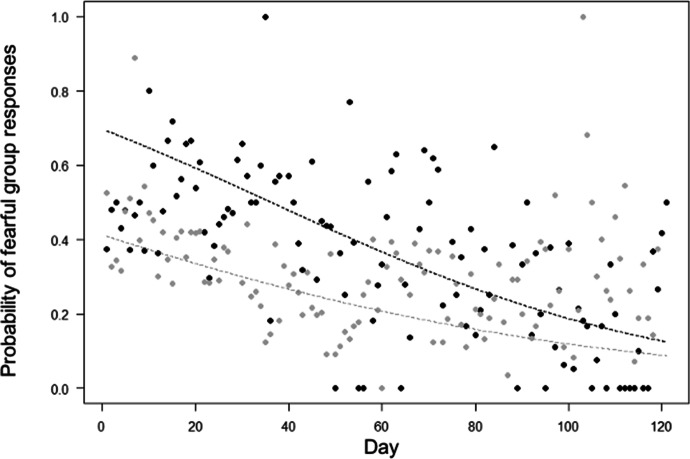
Mean probability that moor macaques in Bengo and Bira on Sulawesi Island, Indonesia (September 2019 to March 2020) displayed fearful group responses during the encounter, as a function of time (i.e. observation day). Indicated is the mean probability of showing fearful responses per observation day (points), and the fitted model (dashed lines). Black represents observations and model line for Merah group, and grey for Scuba group. We aggregated data points per day for clarity, so the model depicted here differs from Model 5 (where encounter number instead of day number was used as test predictor). The model is an interaction plot (between day and group).

Finally, for Model 5, the full-null model comparison was significant (LRT: χ^2^ = 395.50, df = 11, *P* < 0.001). The probability of fearful group responses decreased through time in both groups (in line with Prediction 5; Table I), but more quickly in the Merah than in the Scuba group (in contrast with prediction 10; Table I; Fig. 6). Moreover, fearful group responses were less likely when more researchers were present (in contrast to Prediction 6), when they were in trees (in line with Prediction 7). Fearful group responses varied depending on the area of the encounter (in line with Prediction 8; Tables I and II), being more likely in forest areas (estimate: 0.46) than in areas frequently used by humans (estimate: 0.34), in paths (estimate: 0.40) and beach areas (estimate: 0.23; *P* values for comparisons, respectively: *P* < 0.001; *P* = 0.045; *P* = 0.038). Moreover, fearful responses were less likely when researchers maintained a larger minimum distance to the macaques (in line with Prediction 9; Tables I and II).

## Discussion

In line with previous studies (Blom *et al.,* 2004; Doran-Sheehy *et al.,* 2007; McLennan & Hill, 2010; Samuni *et al.,* 2014; but see Narat *et al.,* 2015; Van Krunkelsven *et al.,* 1999), we found that over the study period (i.e., 7 months) the habituation process led to a significant increase in the proportion of time that researchers spent with the macaques (especially in the group with more previous exposure to humans). However, the daily time required to first locate the macaques did not decrease through time, which contrasts with findings from the only other available study describing the habituation process of moor macaques (Hanson & Riley, 2018). The time required to first locate the macaques, however, can be affected by either the level of macaque’s tolerance to the presence of researchers in the area or by the researchers’ ability to locate the macaques, which in turn can be affected by habitat characteristics (e.g., forest vegetation density, or landscape). Compared with Hanson and Riley’s (2018) field site, our two study sites lack conspicuous karst areas and thus are easier to walk through, which facilitated locating the macaques. This difference in the physical characteristics of the habitats could explain the lower time required to first locate the macaques in this study (i.e., almost half of the time required by Hanson & Riley, 2018). Therefore, it is possible that efficiency at locating the macaques in our study did not increase through habituation, because it was already relatively high from the beginning. In this respect, the results appear to mirror the ecological conditions (and thus the possibility for researchers to locate the macaques), rather than a lack of increase in the macaques’ tolerance to the presence of researchers.

The likelihood of fearful group responses decreased during the study, while the likelihood of neutral group responses increased. Moreover, the mean of minimum distance between the macaques and researchers decreased through time, with a mean distance by the end of the study (i.e., last 10 habituation days) of 14 m in both study groups. A larger minimum distance between researchers and macaques during encounters further favored an increase in neutral group responses and a decrease in fearful ones, suggesting greater tolerance toward the presence of the researchers by the end of the study. Although group responses greatly vary across species, wild animals generally avoid human encounters, responding with alarm calls, threats, or fleeing (Blumstein, 2006; Carrete and Tella, 2017). Throughout the habituation process, where animals were repeatedly exposed to humans, these responses tended to disappear, while neutral responses became more likely, in line with previous studies (Bertolani & Boesch, 2007; Cipolletta, 2003; Hanson & Riley, 2018; McDougall, 2011; Narat *et al.,* 2015; Samuni *et al.,* 2014; Williamson & Feistner, 2011). However, the habituation process does not always lead to a reduction in distance between researchers and animals. For instance, in the previous study on moor macaques conducted at a different study site (Hanson & Riley, 2018), distance between researchers and macaques did not significantly decrease during the habituation period. As discussed previously, these contrasting findings could be due to differences between the field sites, but they also could reflect differences in animals’ response to humans due to factors, such as group composition, differences in individual histories, and experiences with humans. Finally, in our study, we operationalized the minimum distance between researchers and macaques as the minimum distance between researchers and the closest macaque reached during each encounter. This allowed us to record this measure from the beginning of the habituation (when not all macaques could yet be reliably identified). However, future studies should consider measuring the minimum distance between the researchers and the center of the group; this measure might better reflect the response of all individuals in the group to humans. Similarly, future studies could include further measures to assess neutral and fearful responses, including the occurrence of self-directed behaviors, which are a well-established indicator of social tension in primates (Maestripieri *et al.,* 1992).

In contrast to our prediction and to the literature (Williamson & Feistner, 2011), a higher number of researchers facilitated habituation, by increasing the probability of neutral responses and decreasing the probability of fearful ones. It is possible that a higher number of researchers could be detected sooner by the macaques, allowing them to avoid sudden encounters that often lead to fear and flee responses (Crofoot *et al.,* 2010). However, the maximum number of researchers following the macaques in our study was three, and having a higher number of researchers might have negative consequences on the welfare of the macaques by, for instance, changing their feeding habits or response to predators (Kinnaird & O’Brien, 1996; Williamson & Feistner, 2011, for a discussion about the effects of human presence on primate behavior).

Importantly, our findings cannot be explained by the fact that more researchers could more reliably collect detailed behavior in the group. Had it been the case, fearful group responses should have been more easily coded in the presence of more observers (and neutral group responses in the presence of fewer observers), which was instead the opposite of what we found in our study (i.e., the probability of fearful group response decreased with a higher number of researchers, while the probability of neutral group response increased).

When macaques were in trees, they were more likely to respond with neutral behaviors and less likely to respond with fearful ones compared with when they were on the ground, in line with previous studies (Bertolani & Boesch, 2007; Blom *et al.,* 2004; McLennan & Hill, 2010; Narat *et al.,* 2015; Van Krunkelsven *et al.,* 1999; Williamson & Feistner, 2011). However, when macaques were in trees, the minimum distance to the researchers was larger. This can be likely explained by the fact that, when being in trees, macaques might be forced to reach certain heights (e.g., if the tree has no low branches), thus increasing their distance to the researchers (McLennan & Hill, 2010).

The area in which a given encounter took place predicted the probability of neutral and fearful group responses, but not the minimum distance reached during the encounter. In particular, neutral responses increased and fearful responses decreased where visibility was greater (i.e., in beach, path, and human areas compared with forest areas). By reducing the likelihood of sudden contacts, greater visibility might facilitate neutral responses and reduce fear in macaques, ultimately speeding up the habituation process (Ando *et al.,* 2008; Souza-Alves & Ferrari, 2010; Williamson & Feistner, 2011). However, it also is possible that humans use open areas more often than areas with dense vegetation. If so, macaques could be used to encounter humans more often in those areas, even before the habituation started (Bertolani & Boesch, 2007).

We found several differences between the two study groups. These differences only involve two groups and therefore should be taken with caution. In the Merah group, whose previous exposure to humans was more limited, the proportion of time spent with the macaques remained relatively constant through habituation (while it quickly increased in the Scuba group), and the time to first locate the group was generally higher than in the Scuba group. This result is likely due to two main factors. First, the Merah group might have been more cautious towards humans, and habituation might have been slower than for the Scuba group. Second, the Scuba group lived in areas with higher visibility (e.g., beach), which might have facilitated the habituation process. However, in contrast to our predictions, minimum distance between macaques and researchers and fearful group responses decreased more quickly through time in the Merah than in the Scuba group, whereas neutral group responses more quickly increased. Overall, previous exposure to humans might have a more complex effect on primate behavior during the habituation process than we had hypothesized. For instance, it is possible that macaques with less human experience might show a higher initial level of fearful responses, and thus show a steeper decrease in this response through the habituation process, as in our study. Moreover, different kinds of previous exposure to humans (e.g., to hunters or poachers, tourists or local villagers) might predict differences in the way macaques respond to researchers. Apart from their different exposure to humans, the study groups also differed in several other socioecological aspects that might have affected their responses during the habituation (e.g., group size and composition, seasonality). Therefore, it is not possible to infer from these data how macaques with no previous exposure to humans, or under different climatic or ecological conditions, would react to a similar habituation protocol. These might be interesting variables to assess in future studies.

## Conclusions

We described the habituation process of two groups of wild moor macaques over 7 months. The long-term consequences of habituating primates raise ethical concerns that should be carefully considered on a case-by-case basis, before starting habituation, as this process may put wild animals at serious risk (e.g., transmission of COVID-19 and other infectious diseases; Damas *et al.,* 2020; Santos *et al.,* 2020). However, if habituation is considered necessary (e.g., for conservation purposes), previous studies may provide practical hints that might limit the negative shorter-term consequences of the habituation process. In our case, our findings may be especially relevant for researchers working on species with similar ecology and/or behavioral repertoire (e.g., other macaques in Sulawesi). During habituation, in particular, we suggest that researchers should (i) preferentially approach macaques in areas of greater visibility and/or with more trees (as this is less stressful for the groups), and (ii) avoid following macaques alone in the forest (as being alone is less effective and less safe for researchers). Primates react very differently to the presence of humans depending on their individual histories and previous experience to humans, so findings are not easily generalizable (Allan *et al.,* 2020; Ampumuza & Driessen, 2020; Green & Gabriel, 2020). Indeed, moor macaques reacted to our habituation process differently from some other primate species (e.g., in terms of their response to the number of researchers; Johns, 1996). Therefore, the habituation of wild animals for research purposes should be tailored to species-specific ecology, behavior, and cognition to minimize the risks that this process might have for the animals’ welfare.
